# Occurrence and Quantitative Risk Assessment of Twelve Mycotoxins in Eggs and Chicken Tissues in China

**DOI:** 10.3390/toxins10110477

**Published:** 2018-11-16

**Authors:** Lan Wang, Qiaoyan Zhang, Zheng Yan, Yanglan Tan, Runyue Zhu, Dianzhen Yu, Hua Yang, Aibo Wu

**Affiliations:** 1 SIBS-UGENT-SJTU Joint Laboratory of Mycotoxin Research, CAS Key Laboratory of Nutrition, Metabolism and Food Safety, Shanghai Institute of Nutrition and Health, Shanghai Institutes for Biological Sciences, University of Chinese Academy of Sciences, Chinese Academy of Sciences, Shanghai 200000, China; wanglan@sibs.ac.cn (L.W.); zyan@sibs.ac.cn (Z.Y.); tyl2721@hotmail.com (Y.T.); runyue.zhu@outlook.com (R.Z.); dzyu@sibs.ac.cn (D.Y.); 2 Institute of Quality and Standard for Agro-Products, Zhejiang Academy of Agricultural Sciences, Hangzhou 310021, China; yanyan0014@163.com

**Keywords:** risk assessment, co-occurring mycotoxins, eggs, chicken tissues

## Abstract

Aflatoxins (AFs), deoxynivalenols (DONs), and zearalenones (ZENs) are common mycotoxins that contaminate feedstuff, causing contamination of poultry products. In our study, these mycotoxins were quantified in 152 egg samples collected from markets in Jiangsu (JS), Zhejiang (ZJ), and Shanghai (SH) and in 70 chicken tissue samples (liver, heart, and gizzard) from ZJ in China. The main mycotoxins observed in egg samples were DON, 15-AcDON, and ZEN, although only ZEN family mycotoxins (ZEN, α-ZEL, β-ZEL, and α-ZAL) were detected in chicken tissues. Furthermore, for the first time, we assessed the health risks of exposure of three populations (children, adults, and elder adults) to DONs (DON, 3-AcDON, and 15-AcDON) and ZEN in eggs (from three different areas) and to ZEN in chicken tissues. We show that the mean dietary intake (DI) values and the 97.5th percentile DI values of DON and ZEN through egg ingestion were lower than the provisional maximum tolerable daily intake (PMTDI) (1 μg/kg body weight (BW)/day) for the three populations in the three geographical areas studied. However, eggs contaminated with high levels of DONs and ZEN contributed to a large proportion of the PMTDI of these mycotoxins, especially in children and elder adults. Although ZEN was highly detected in the chicken tissues, no significant health risk was observed.

## 1. Introduction

Mycotoxins are natural small-molecule contaminants produced as secondary metabolites by fungi [1]. It has been reported that approximately one-fourth of the world’s crops are contaminated by mycotoxins [2]. Contamination by mycotoxins occurs frequently in chicken feed, including maize and other cereals [3]. Mycotoxins are relatively stable and readily enter animals and humans, causing various toxic effects [4]. In recent decades, mycotoxins have attracted increasing attention worldwide because of their frequent occurrence [5]. 

Among mycotoxins, aflatoxins (AFs), produced by *Aspergillus flavus* and *Aspergillus parasiticus* molds, are classified as the most toxic. The evaluation of epidemiological and laboratory results demonstrated that there is sufficient evidence in humans for the carcinogenicity of naturally occurring mixtures of aflatoxins, which have also been classified as a group I carcinogen by an international agency [6,7]. Type B trichothecenes have been associated with a spectrum of toxic effects in animals. To date, the most frequently found mycotoxins are deoxynivalenol (DON) and its acetylated derivatives, 3-acetyldeoxynivalenol (3-AcDON) and 15-acetydeoxynivalenol (15-AcDON) [8]. DON causes acute human diseases such as nausea, vomiting, gastrointestinal upset, and diarrhea [9]. As reported, acetylated DONs show stronger toxicity, because these toxins are more rapidly absorbed into the intestine [10]. Humans can easily be exposed to DON, and dietary intake is considered to be a major pathway. In most countries, DON is detectable in cereals. For example, 89.3% of wheat samples in Jiangsu Province in China were contaminated with DON in concentrations ranging between 259 and 4.98 × 10^3^ μg/kg, with a mean of 1.96 × 10^3^ μg/kg [11]. Importantly, DON can persist in eggs and meat after poultry animals are fed with contaminated feed. Consequently, the previous provisional maximum tolerable daily intake (PMTDI) of 1 μg/kg body weight (BW)/day [12] of DON was extended to a PMTDI for the three compounds together by JECFA in 2010 [13]. Zearalenone (ZEN) and its metabolites have been reported as common food contaminants, especially in cereals [14]. The most important toxic effect of the ZEN family is its estrogenic effect [15], which has been found in children with precocious sexual development who were exposed to contaminated food [16]. ZEN in organ tissues has been studied in livestock, but limited information is available, particularly for broiler chicken [17,18]. Due to the high toxicity of ZEN, a PMTDI of 0.5 μg/kg BW/day was established in 2001 [13]. 

Recently, liquid chromatography–tandem mass spectrometry (LC-MS/MS) has been extensively used as a highly selective and sensitive analytical method for mycotoxin determination in feed, eggs, and animal tissues [19,20]. The quick, easy, cheap, effective, rugged, and safe (QuEChERS) method, prior to LC-MS/MS, was a widely used sample preparation methodology that was successfully applied to detect various mycotoxins [21,22]. In our previous studies, we developed a simple pretreatment method for multimycotoxin determination in eggs and chicken tissues by liquid chromatography–tandem mass spectrometry [23,24]. These studies demonstrated that QuEChERS is a suitable pretreatment for the simultaneous extraction of multimycotoxins from eggs and different tissues.

Various surveys have been conducted to investigate the presence of mycotoxins in feed. A three-year worldwide survey reported that 48% of 7045 analyzed feedstuff samples were contaminated by two or more mycotoxins [25]. Importantly, recent analytical results from animal feed samples obtained between 2007 and 2012 showed higher levels of DON in poultry feed [26]. For example, Palacios (2017) documented that DON was found in all analyzed samples at concentrations varying between 50 μg/kg and 9.48 × 10^3^ μg/kg [27]. Chickens fed contaminated feed can show toxic effects such as fatty liver, kidney disorders, and leg and bone problems. Therefore, eggs and chicken meat can potentially have food safety issues for humans [28,29]. There are few studies dealing with the transmission of AF [30], DON [31,32], and ZEN [33] to poultry products, including eggs and tissues. Although the transmission levels were found to be low in these studies, several reports have documented the presence of AF, DON, or ZEN in eggs and chicken meat [3,21,34,35]. Tangni et al. reported that home-produced eggs may contribute 1.0% of the PMTDI (1 μg/kg BW/day) for human exposure to DON. The detection of such high levels of mycotoxins in eggs as well as the frequent contamination of chicken meat justify screening a larger number of eggs and tissues over a wider geographical area, particularly in China, where chicken eggs are an important contributor to the human diet [36]. As little information about mycotoxins in poultry products is available in China, it is necessary to evaluate the occurrence of multiple mycotoxins in those products.

In general, risk assessment is the systematic characterization of potential adverse effects caused by exposure to hazardous agents. Numerous papers have reported that the human population is exposed to low doses of mycotoxins through food consumption, especially cereals and cereal products [37,38,39]. However, there is little information about the risk of exposure to AFs, DONs, and ZENs by consuming poultry products in China. The present study investigated the levels of 12 mycotoxins including AFs, DONs, and ZENs in eggs from three different areas, Jiangsu, Zhejiang, and Shanghai, and chicken meat from Zhejiang. Additionally, a quantitative dietary exposure assessment of mycotoxins was conducted using the contamination data to determine exposure estimates within the three populations (children, adults, and elder adults). The results of our study are helpful to raise awareness of the health risks associated with these toxins. 

## 2. Results and Discussion

### 2.1. Occurrence of Mycotoxins in Egg Samples

**Figure 1:**
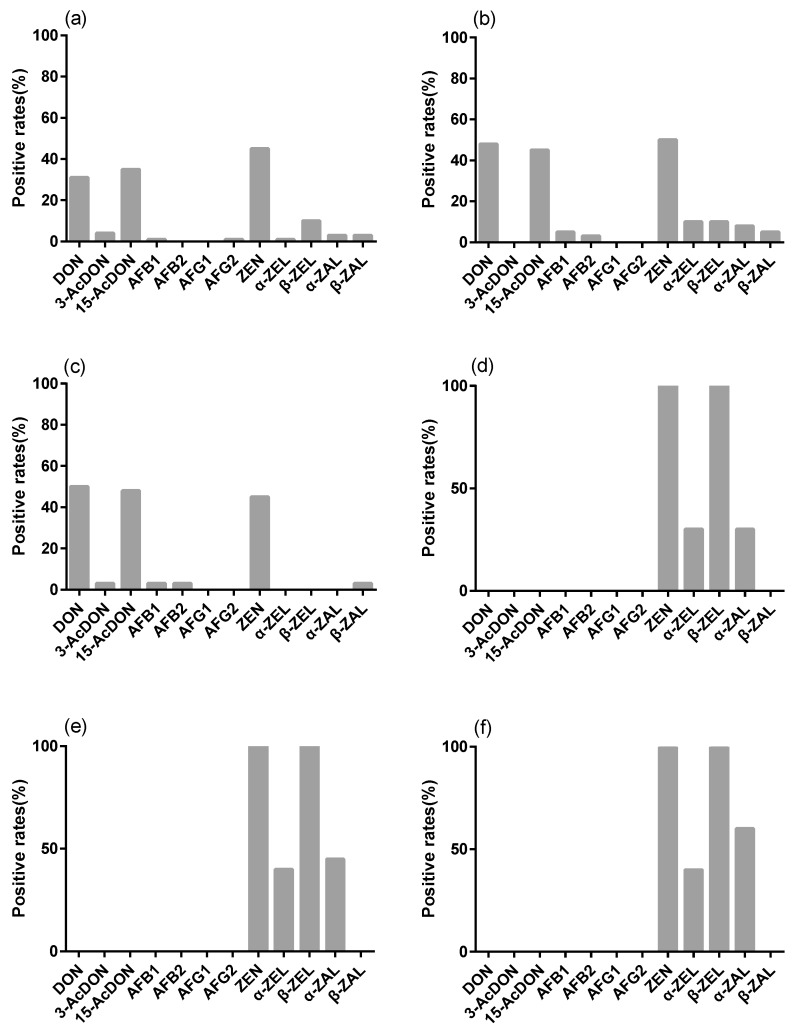
Proportions of samples positive for mycotoxins. (**a**) Eggs from Jiangsu (JS) (*n* = 72); (**b**) eggs from Zhejiang (ZJ) (*n* = 40); (**c**) eggs from Shanghai (SH) (*n* = 40); (**d**) liver tissue from chickens (*n* = 30); (**e**) heart tissues from chickens (*n* = 20); (**f**) gizzard tissues from chickens (*n* = 20).

In the present study, 152 samples of eggs were screened for the presence of 12 mycotoxins. Table 1 shows the mean and median concentrations of those mycotoxins in egg samples from different Chinese areas. The proportions of samples positive for the mycotoxins are shown in Figure 1. The most frequent mycotoxins observed in the egg samples were DON, 15-AcDON, and ZEN. AFB_1_, AFB_2_, and AFG_2_ were rarely detected in egg samples and AFG_1_ was never detected. The highest incidences of DON, ZEN, and 15-AcDON were noticed in egg samples from Shanghai (SH). Mycotoxin contamination in eggs could be attributed to feed quality [40]. Thus, the presence of mycotoxins in eggs may reflect mycotoxin contamination of the feed.

#### 2.1.1. Level of Contamination by Mycotoxins in Egg Samples from Jiangsu

The results of 72 egg samples from Jiangsu (JS) showed that 31% tested positive for DON, with an average of 96.2 μg/kg (median value = 8.63 μg/kg). In a previous report, a lower range of DON concentration (0.6–17.9 μg/kg) was found in home-produced eggs in Belgium [34]. The median value of DON in our samples was a little higher than that in the study by Tangni et al. (2.0 μg/kg and 3.0 μg/kg in eggs collected in autumn and spring). The DON contamination level was slightly lower than that in wheat in China (0.02–5.15 × 10^4^ μg/kg) [41] and in Argentina (<50–9.48 × 10^3^ μg/kg). The acetylated forms, 15-AcDON and 3-AcDON, were also present in 35% and 4% of the samples, with mean concentrations of 43.6 and 42.9 μg/kg and medians of 10.5 and 22.8 μg/kg, respectively. The results show that the 3-AcDON contamination level was between 16.8 and 89.1 μg/kg, but it rarely occurred. A relative ZEN contamination level of 44%, with a range of 0.30–418 μg/kg, was detected in egg samples in JS. The average level of ZEN was found to be 29.1 μg/kg (median value = 17.0 μg/kg), higher than the results from the previous study [3]. Iqbal et al. reported that 32% of eggs (*n* = 80) collected in Pakistan were found to be positive for ZEN at a concentration of 1.58 μg/kg [3]. Meanwhile, our results show that the ZEN family mycotoxins, including α-ZEL, β-ZEL, α-ZAL, and β-ZAL, were also detected with concentrations of 4.28, 4.41, 0.86, and 1.00 μg/kg, respectively. Among the AFs, AFB_1_ was found in only one egg sample, at a concentration of 168 μg/kg, which is higher than the findings (0.85–2.41 μg/kg) in the study by Iqbal et al. [3], while AFG_2_ was found in one sample at a low concentration of 1.47 μg/kg. Amirkhizi et al. found that 58% of egg samples from Iran were contaminated with AFB_1_ at 0.30–16.4 μg/kg [42]. The proportion of egg samples positive for AFB_1_ in their study was higher than that in our study.

#### 2.1.2. Level of Contamination by Mycotoxins in Egg Samples from Zhejiang 

DON and 15-AcDON were detected in the Zhejiang (ZJ) samples, with an average of 44.0 and 29.5 μg/kg, respectively, which are higher than the results from a previous study (0.6–17.9 ng/g) [34]. In addition, the median value of DON in eggs from ZJ was much higher than that in a previous study in Belgium [34]. 3-AcDON was not detected in ZJ egg samples. ZEN, α-ZEL, β-ZEL, α-ZAL, and β-ZAL levels in the eggs from ZJ were found to be at concentrations of 29.7, 13.4, 3.93, 4.86, and 2.88 μg/kg. These values are much higher than those found in similar products in Belgium [34], in which trace levels (<limit of quantification (LOQ)) were observed in most (6–9 out of 10) egg samples collected, especially in the autumn. In spite of this difference in concentration level, the proportion of egg samples positive for ZEN in the study of Tangni et al. was similar to that found in the present study. Concerning AFB_1_, only one sample was detected, with a concentration of 4.58 μg/kg. This value was in the range of that reported by Amirkhizi et al. (0.30–16.4 μg/kg) [42] and slightly higher than that reported by Iqbal et al. (0.85–2.41 μg/kg) [3]. 

#### 2.1.3. Level of Contamination by Mycotoxins in Egg Samples from SH

In the present study, 40 eggs from SH were analyzed for the presence of the 12 mycotoxins. DON, 15-AcDON, and ZEN were the most commonly detected mycotoxins in the samples, with positive rates of 50%, 48%, and 45%. All the DONs—DON, 15-AcDON, and 3-AcDON—were detected in the egg samples, with medians of 45.9, 51.5, and 5.94 μg/kg, respectively. Among the ZEN metabolites, only β-ZAL was detected in one sample at a concentration of 0.35 μg/kg. AFB_1_ was found in only one sample at a concentration of 1.46 μg/kg, a concentration similar to those found in previous studies [3,35]. Indeed, AFs were found with a mean of 1.23 μg/kg in 10 eggs by Herzallah [35] and a mean of 1.39 μg/kg in 80 eggs by Iqbal [3].

### 2.2. Occurrence of Mycotoxins in Tissue Samples

In the present study, a total of 70 chicken tissue samples, including liver, heart, and gizzard tissues, were analyzed for the occurrence of 12 mycotoxins. The results in Figure 1 and Table 2 show that four ZEN family mycotoxins, ZEN, β-ZEL, α-ZEL, and α-ZAL, were detected in the tissues, while the mean concentration of α-ZAL was <LOQ (2 μg/kg). All chicken tissue samples were found to be positive for ZEN and β-ZEL. The results were in agreement with the finding that ZEN might have the ability to infiltrate the various tissues of broiler chickens [18]. The proportions of samples positive for ZEN mycotoxins were found to be similar in the three types of chicken tissues, while the levels in gizzard were slightly higher than those in liver and heart. AFs and DONs were not detected in the tissues. Our findings are similar to those of Dänicke, in which DON concentration was also lower than the detection limits of 2 ng/mL (plasma of turkeys) and 4 μg/kg (liver of turkeys) [43]. 

#### 2.2.1. Level of Contamination by Mycotoxins in Chicken Liver Samples 

The results in Table 2 show that the ZEN mycotoxins ZEN, β-ZEL, and α-ZEL were detected in the liver samples at mean concentrations of 62.9, 20.1, and 2.32 μg/kg, respectively. Such high levels of contamination of broiler livers by ZEN have never been previously described; Iqbal et al. reported levels as low as 3.45 ± 0.57 μg/kg [3]. AFs and DONs were not detected in the liver samples. 

#### 2.2.2. Level of Contamination by Mycotoxins in Chicken Heart Samples 

Similar results were obtained in the heart samples (Table 2). The mean levels of ZEN, β-ZEL, and α-ZEL in the heart samples were 64.6, 17.7, and 3.60 μg/kg, respectively. All the heart samples were found to be positive for ZEN and β-ZEL. Only 40% and 45% of samples were positive for α-ZEL and α-ZAL. AFs and DON were not detected in these samples.

#### 2.2.3. Level of Contamination by Mycotoxins in Chicken Gizzard Samples 

All the gizzard samples tested were positive for ZEN and β-ZEL; 40% and 60% were contaminated by α-ZEL and α-ZAL, respectively. The average levels of ZEN, β-ZEL, and α-ZEL in the samples were 71.6, 25.0, and 4.01 μg/kg, respectively, which are a little higher than those of the liver and heart samples.

### 2.3. Risk Assessment for Eggs

The most common mycotoxins detected in eggs from the three Chinese regions were DON, 15-AcDON, and ZEN. As reported, acetylated DONs showed stronger toxicity because these toxins are more rapidly absorbed into the intestine [10], thus the risk of exposure to DONs (DON, 3-AcDON, and 15-AcDON) by ingesting eggs was estimated in our study. To date, regulatory limits have only been set up for ZEN, but not for its modified forms, so only the risk of exposure to ZEN was estimated in our study.

#### 2.3.1. Point Evaluation

Intake of mycotoxins from eggs was estimated for consumers by multiplying egg consumption data normalized to the average body weight of the target population by the mycotoxin contents obtained in this study. This provides a realistic and appropriate estimation for each specific group—children, adults, and elder adults—which can be compared to the PMTDI values. The obtained values are shown in Table 3. 

Based on the habitual average intake of eggs from a national representative food intake survey, intake of DONs from egg consumption was estimated to reach to about 5.2%, 3.9%, and 5.7% of the PMTDI (1 μg/kg BW/day) for children in JS, ZJ, and SH, respectively. The dietary intake (DI) values for children living in JS, ZJ, and SH in the 97.5th percentile were estimated to be 0.291, 0.222, and 0.194 μg/kg BW/day, respectively. In other words, the mean margin of safety (MOS) values and the 97.5th percentile MOS values were all less than 1, suggesting that no obvious health risks were observed for children. Nevertheless, the maximum DI value (2.34 μg/kg BW/day) obtained for the JS region was above the PMTDI (1 μg/kg BW/day), suggesting that adverse effects might occur in children if they consume eggs highly contaminated by DONs. In the present study, means of 2.2%, 1.6%, and 2.4% of the PMTDI (1 μg/kg BW/day) for adult exposure to DON intake from eggs were observed. For adults living in JS, ZJ, and SH, the DI values at the 97.5th percentile of DON intake from eggs were 0.121, 0 0.092, and 0.081 μg/kg BW/day, respectively. Furthermore, the maximum intake of DONs estimated for adults was 0.968 (JS), 0.124 (ZJ), and 0.105 (SH) μg/kg BW/day, respectively. When eggs are highly contaminated with DONs, they might contribute a large proportion of the provisional maximum tolerable daily intake, which is hazardous for health. We assessed the DI values of DONs in elder adults and identified the values due to contaminated egg consumption. We found that the highest DI value was 1.09 μg/kg BW/day in JS. This maximum value was slightly higher than the PMTDI (1 μg/kg BW/day). Overall, our findings suggest that intake of DONs via egg consumption might occur rarely in the three geographical regions. However, DON intake for children consuming highly contaminated eggs from Jiangsu was found to be higher than the PMTDI (1 μg/kg BW/day). Therefore, urgent steps should be taken to monitor and control these toxins in eggs. 

The results of the risk assessment of ZEN in eggs from the different areas, performed by point evaluation, are presented in Table 4. The DI values of ZEN via egg consumption estimated for the three populations were found to be lower than the PMTDI (0.5 μg/kg BW/day). According to the data from Jiangsu, means of 4.2%, 1.8%, and 1.8% of the PMTDI (0.5 μg/kg BW/day) for eggs were established for children, adults, and elder adults, respectively. For consumers in Zhejiang, means of 5.4%, 2.2%, and 2.6% of the PMTDI (0.5 μg/kg BW/day) were found. Furthermore, the results demonstrate that eggs may account for 6.2%, 2.6%, and 2.8% of the PMTDI (0.5 μg/kg BW/day) for human exposure to ZEN on average in SH. The maximum intake of ZEN estimated for children was 0.853 μg/kg in Zhejiang, which is higher than the PMTDI (0.5 μg/kg BW/day). These results show that children are more sensitive to ZEN than adults and elder adults. As children are sensitive to mycotoxin levels, it is worthwhile to pay attention to feed quality as well as the environment during the transportation and storage of eggs.

#### 2.3.2. Monte Carlo Assessment Model

Point evaluation might give an overevaluation of the exposure without considering the variability and uncertainty of food consumption and contamination levels. Therefore, a full probabilistic method, the Monte Carlo model, was used for further investigation to provide more realistic estimates of exposure. The DI values at the mean, 50th, 60th, 70th, 80th, 90th, and 95th percentiles are shown in Table A1. All the DI values of eggs from different areas for the three populations were below the PMTDI (1 μg/kg BW/day for DONs, 0.5 μg/kg BW/day for ZEN), meaning that the MOS values were less than 1. Intake of DONs and ZEN was estimated by the Monte Carlo assessment model, which showed that eggs may contribute 16.2% and 18.3% of the PMTDI (1 μg/kg BW/day, 0.5 μg/kg BW/day) at the 95th percentile for children. Based on the samples analyzed, we estimate that eggs are not a major route of DON and ZEN exposure in China. 

### 2.4. Risk Assessment for Chicken Tissues

The systematic detection of ZEN in chicken tissues has raised the issue of exposure for consumers. Several studies have demonstrated that ZEN and its main metabolites have anabolic activity in farm animals, which causes serious health problems [44,45]. However, in contrast to ZEN, no regulatory limits have been set for its modified forms. Therefore, a risk assessment of consumers exposed to only ZEN through chicken consumption was evaluated in our study. The results obtained by the point evaluation method are shown in Table 5. The mean DI values, the 97.5th percentile, and the maximum DI values of ZEN due to contaminated chicken consumption by the three populations were found to be much lower than the PMTDI (0.5 μg/kg BW/day), which means that all MOS values were less than 1. These findings suggest that chicken consumption contributes very little to consumer exposure, even if high levels of ZEN were found in those tissues. Based on the levels of ZEN in the samples, chicken tissues are estimated to contribute a maximum of 2.4%, 1.6%, and 1.2% of the PMTDI (0.5 μg/kg BW/day) for children, adults, and elder people, respectively. The DI values for children were higher than those for adults and elder adults. Similarly, the DI values (Table A2) at the mean, 50th, 60th, 70th, 80th, 90th, and 95th percentiles were much lower than the PMTDI (0.5 μg/kg BW/day), suggesting that no significant health risks for the three populations are expected. Nevertheless, as only ZEN was considered in this model, negative health effects due to the combined exposure to ZEN and its modified forms cannot be excluded.

## 3. Conclusions

Food safety and security constitute a basic human need. Ensuring the safety of food is a major focus of international and national action. Human beings are exposed to mycotoxins through a variety of routes. The routes of exposure include oral intake (water and food), inhalation (environment), and dermal penetration (environment). The most frequent and important route of exposure to mycotoxins is the consumption of contaminated cereal-based and animal-derived food. Indeed, mycotoxins in animal feed could be carried over into the animal tissues, particularly liver, kidney, and eggs [33]. 

Little data has been obtained on the transmission of mycotoxins from feed to eggs and tissues. Prelusky et al. estimated that 0.31% of DON in feed goes to eggs [46]. In addition, Sypecka et al. (2004) stated that the transmission of ZEN and its metabolites from contaminated feed to eggs was below 0.3% [33]. However, several studies detected these mycotoxins in eggs and tissues. For example, Makun et al. showed that 85% of tested eggs were contaminated by DON at concentrations ranging from 0.6 to 17.9 ng/g [30]. Recently, Xu et al. researched DON levels in chicken tissues from Guangzhou, China [47]. Among those samples (*n* = 20), DON was present in one muscle (2.1 μg/kg) and two kidney (1.3–2.0 μg/kg) tissue samples. 

As eggs and chickens are consumed widely in China, it is important to evaluate the risk of exposure by consumers to the mycotoxins that are frequently present in them. In our study, a total of 152 samples of eggs from three Chinese regions, Jiangsu, Zhejiang, and Shanghai, were screened for the presence of three populations of mycotoxins, AFs, DONs, and ZENs. We found that AFs were rarely present in eggs and absent in tissues. DON, 15-AcDON, and ZEN were frequently present in egg samples, although only ZENs were detected in tissue samples. Therefore, we assessed the health risks of consumption of eggs and tissues contaminated by DONs and ZEN by three populations, children, adults, and elder adults, living in three areas, JS, ZJ, and SH. The risk assessments were estimated by a point evaluation and the Monte Carlo model. Both models suggested that eggs and tissues contribute a small amount to the mycotoxin intake. Nevertheless, when eggs might be contaminated with high levels of mycotoxins, this contributes to a large proportion of the provisional maximum tolerable daily intake for DONs and ZEN. 

However, in some extreme cases, such as large consumption of eggs (e.g., by pregnant and postpartum women and patients who take protein supplements), there may be some health problems. To conclude, the results of our study provide evidence supporting the idea that regulatory limits of ZENs and DONs in eggs should be implemented. Strict control of chicken feed and environment (production, transportation, and storage) are important in reducing the risks to human health.

## 4. Materials and Methods

### 4.1. Chemicals and Apparatus

The AFB_1_, AFB_2_, AFG_1_, DON, 3-AcDON, 15-AcDON, ZEN, β-ZEL, α-ZEL, β-ZAL, and α-ZAL standard solutions were purchased from Sigma-Aldrich (St. Louis, MO, USA) and stored at −20 °C before use. All organic solvents, salts, and acids were of analytical or HPLC grade. Acetonitrile, methanol, hexane, and isopropanol were purchased from Merck (Darmstadt, Germany). Ammonium acetate and formic acid were purchased from Sigma-Aldrich (St. Louis, MO, USA). Milli-Q quality water (Millipore, Billerica, MA, USA) was used throughout the experiments. An Agilent Extend-C18 column (100 mm × 4.6 mm, 3.5 μm) was obtained from Agela Technologies (Tianjin, China).

### 4.2. Preparation of Standard Solutions

The standards were accurately weighed and dissolved in pure acetonitrile to stock solutions at the following concentrations: 0.5 μg/mL (DON), 1 μg/mL (3-AcDON), 0.6 μg/mL (15-AcDON), 0.1 μg/mL (AFB_1_), 0.1 μg/mL (AFB_2_), 0.1 μg/mL (AFG_1_), 0.1 μg/mL (AFG_2_), 0.5 μg/mL (ZEN), 1.25 μg/mL (α-ZEL), 1.25 μg/mL (β-ZEL), 1.25 μg/mL (α-ZAL), and 1.25 μg/mL (β-ZAL). Then they were serially diluted with acetonitrile to obtain the different working solutions. All working solutions were prepared prior to analysis.

### 4.3. Samples

All the samples were collected from East China. In the text, we defined Jiangsu, Zhejiang, and Shanghai as JS, ZJ, and SH. The chicken heart (*n* = 20), liver (*n* = 30), and gizzard (*n* = 20) samples were randomly obtained from ZJ between 2017 and 2018. The blank samples (heart, spleen, and gizzard) were obtained from local supermarkets in Shanghai and were verified to be toxin-free. Each sample was cut into 10 g slices and stored at −80 °C until analysis (within 2 weeks). A total of 152 eggs were randomly purchased from supermarkets and local markets in Jiangsu (72), Zhejiang (40), and Shanghai (40) between 2015 and 2016. Each sample was stored in an icebox during transport and stored at 4 °C before further treatment (within 2 days). Broken eggs were pooled in a container, mixing the yolk and albumen with a stirring stick. The mixture was transferred into a sterile container. These samples were stored at −80 °C until analysis (within 2 weeks). 

### 4.4. Determination of Multimycotoxins in Eggs

**Figure A1:**
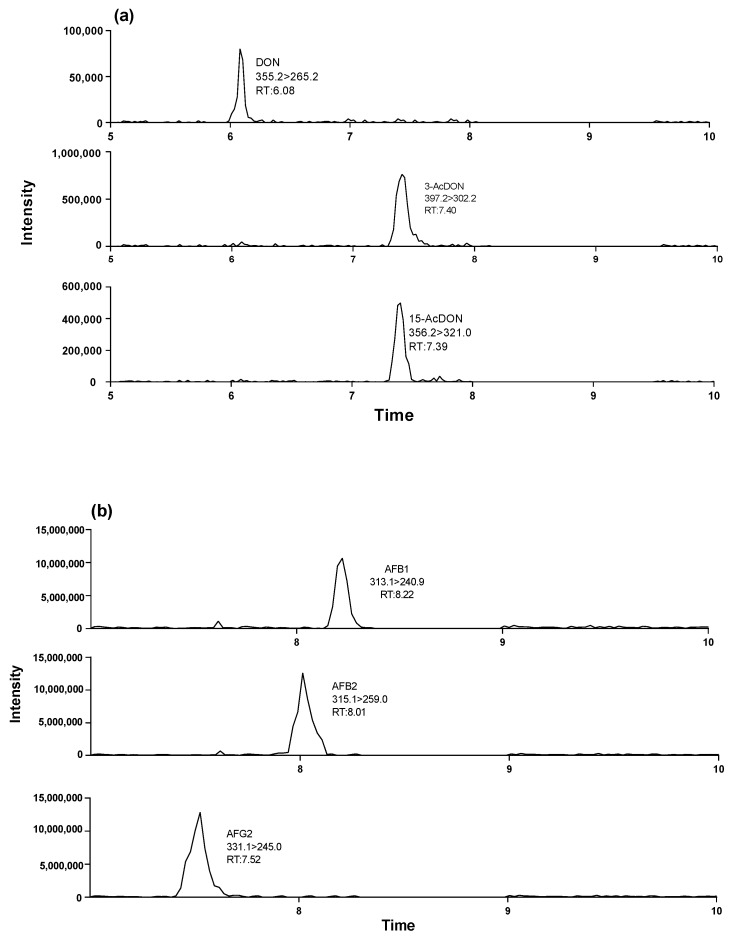

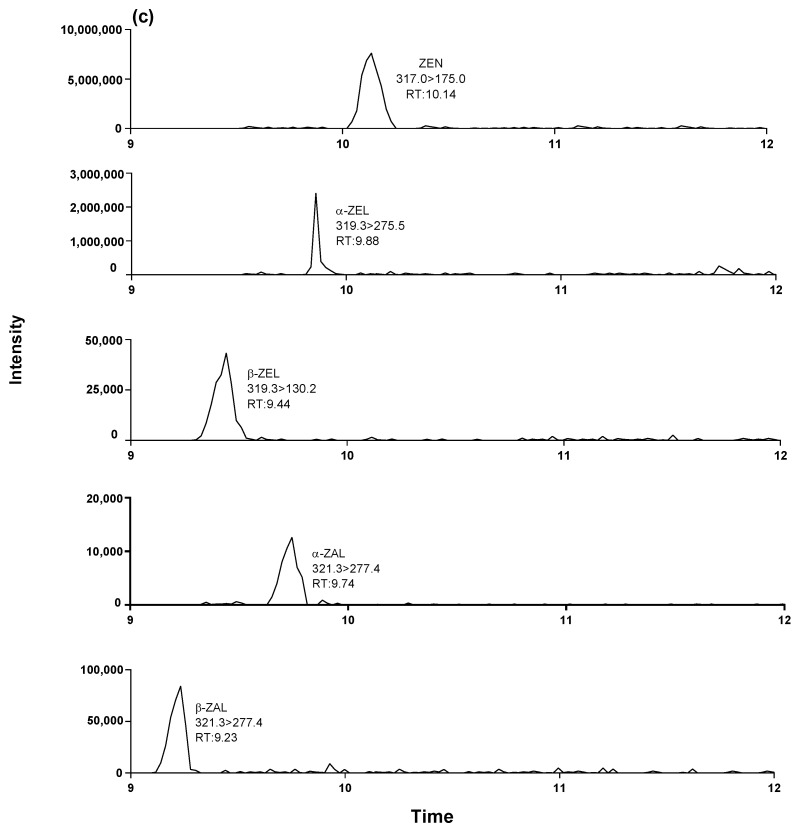
Naturally contaminated egg samples: (**a**) DON (14.28 μg/kg), 3-AcDON (22.82 μg/kg), 15-AcDON (8.33 μg/kg); (**b**) AFB_1_ (1.46 μg/kg), AFB_2_ (1.89 μg/kg), AFG_2_ (1.47 μg/kg); (**c**) ZEN (51.25 μg/kg), α-ZEL (6.51 μg/kg), β-ZEL (5.23 μg/kg), α-ZAL (3.35 μg/kg), β-ZAL (2.88 μg/kg). (RT: Retention Time)

The mycotoxins in eggs were quantified by the QuEChERS-based LC-MS/MS method validated previously [23] and applied in this study. The limit of detection (LOD) for ZENs was 0.1 μg/kg, and the limit of quantification (LOQ) was 0.2 μg/kg. The LODs for AFB_1_, AFB_2_, AFG_1_, and AFG_2_ were 0.1, 0.5, 0.1, and 0.5 μg/kg, and the LOQs were 0.2, 1, 0.2, and 1 μg/kg, respectively. The obtained LODs of the DONs varied from 1 μg/kg to 2 μg/kg, and the LOQs were between 2 μg/kg and 5 μg/kg. A positive sample was defined as having a concentration of mycotoxins above LOD values. If the concentration of mycotoxins in the samples was less than LOD values, this was referred to as not detected (ND). The chromatogram of egg samples containing AFs, DONs, and ZENs is shown in Figure A1.

### 4.5. Determination of Multimycotoxins in Chicken Tissues 

**Figure A2:**
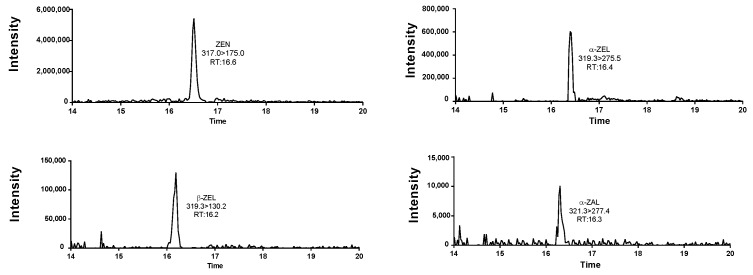
Naturally contaminated chicken tissue samples: ZEN (39.90 μg/kg), α-ZEL (2.16 μg/kg), β-ZEL (18.82 μg/kg), and α-ZAL (3.15 μg/kg). (RT: Retention Time)

Mycotoxins in chicken tissues were quantified by the LC-MS/MS approach. The LOD and LOQ of the DONs were 2 and 5 μg/kg, respectively, while those of the other mycotoxins were 1 and 2 μg/kg. The chromatogram of chicken tissue samples containing ZENs is presented in Figure A2. More specific information about the method applied in our study is in the Appendix A.

### 4.6. Risk Assessment

The risk of exposure by ingesting ZEN and DONs in eggs and chicken tissues was assessed for the 3 human populations by 2 mathematical models: the point evaluation model and the Monte Carlo assessment model, both based on the following equation:

*y* = *x* × *c*/*w* (1)

where *y* is the dietary intake (DI) value of the mycotoxin (μg/kg BW/day), *x* is the mean consumption of the food (g/day), *c* refers to the concentration level of mycotoxin in food (mg/kg), and *w* is body weight (kg). In the present study, non-detected refers to values lower than LOD values. The non-detected values were defined as half of LOD (LOD/2) for the mycotoxin dietary exposure assessment based on the criteria published by Scoop (2004) [48]. The *x* and *w* values used in the model come from data published by the Chinese Nutrition Society and Zhai et al. [49] and are shown in Table 6. 

We calculated risk assessments for the following three human populations: children, adults, and elder adults. The consumption of eggs and chicken tissues, different from the consumption of cereals and cereal-based food, is low. In the present study, the habitual average intake of these food groups was estimated from a national representative food intake survey [49]. Therefore, the mean dietary consumption values of eggs and chicken tissues were considered to be fixed, and the estimated intake of mycotoxins was determined by multiplying the mean food-consumption data (divided by body weight) for the three populations by the key distributions of the mycotoxin exposure data. The Monte Carlo assessment model combined the mean food-consumption data with the distributions of mycotoxins in the food, which involved a scenario in which the levels of mycotoxins were modeled as distributions. The @RISK software package, version 7.0 (Palisade, Ithaca, NY, USA), in combination with Microsoft Excel 2016 was used to run a simulation of 10,000 iterations to perform the Monte Carlo assessment. Then, the values of DI under different percentiles of intake (50th, 60th, 70th, 80th, 90th, and 95th) were consecutively obtained and compared to the values of PMTDI. The risk assessment was evaluated by the margin of safety (MOS) value, which was calculated as follows:

MOS = *y*/PMTDI (2)

The PMTDI values of the DONs and ZEN were 1 μg/kg BW/day and 0.5 μg/kg BW/day, respectively, according to JECFA. The health risk of a mycotoxin is indicated if MOS ≥ 1. Otherwise, there is no significant risk observed.

## Figures and Tables

**Table 1:** Contamination levels of 12 mycotoxins in eggs from different areas (unit: μg/kg). JS, Jiangsu; ZJ, Zhejiang; SH, Shanghai; DON, deoxynivalenol; AFB/AFG, aflatoxin family; ZEN/ZEL/ZAL, zearalenone family; ND, not detected.

Toxin	Eggs from JS			Eggs from ZJ			Eggs from SH		
	Range	Mean	Median	Range	Mean	Median	Range	Mean	Median
DON	2.01–1.60 × 10^3^	96.2	8.63	4.68–135	44	31.8	<2–88.9	43	45.9
3-AcDON	16.8–89.1	42.9	22.8	ND	ND	ND	5.94	5.94	5.94
15-AcDON	<5–664	43.6	10.5	<5–152	29.5	15.3	12.7–155	62.4	51.5
AFB1	168	168	168	2.60–6.55	4.58	4.58	1.46	1.46	1.46
AFB2	ND	ND	ND	1.89	1.89	1.89	1.25	1.25	1.25
AFG1	ND	ND	ND	ND	ND	ND	ND	ND	ND
AFG2	1.47	1.47	1.47	ND	ND	ND	ND	ND	ND
ZEN	0.30–418	29.1	17.0	0.25–986	29.7	22.8	1.54–390	33.3	28.0
α-ZEL	4.28	4.28	4.28	1.88–39.8	13.4	5.87	ND	ND	ND
β-ZEL	0.87–14.4	4.41	2.05	1.29–5.89	3.93	4.27	ND	ND	ND
α-ZAL	0.49–1.22	0.86	0.86	1.07–10.2	4.86	3.35	ND	ND	ND
β-ZAL	0.96–1.03	1.00	1.00	1.94–3.81	2.88	2.88	0.35	0.35	0.35

**Table 2:** Levels of 12 mycotoxins in chicken tissues (unit: μg/kg).

Toxin	Liver			Heart			Gizzard		
	Range	Mean	Median	Range	Mean	Median	Range	Mean	Median
DON	ND	ND	ND	ND	ND	ND	ND	ND	ND
3-AcDON	ND	ND	ND	ND	ND	ND	ND	ND	ND
15-AcDON	ND	ND	ND	ND	ND	ND	ND	ND	ND
AFB1	ND	ND	ND	ND	ND	ND	ND	ND	ND
AFB2	ND	ND	ND	ND	ND	ND	ND	ND	ND
AFG1	ND	ND	ND	ND	ND	ND	ND	ND	ND
AFG2	ND	ND	ND	ND	ND	ND	ND	ND	ND
ZEN	40.0–74.0	62.9	64.8	49.3–87.5	64.6	61.5	39.9–84.9	71.6	75.1
α-ZEL	<2–7.10	2.32	<2	<2–9.60	3.60	<2	<2–10.4	4.01	<2
β-ZEL	11.8–28.3	20.1	20.1	9.41–27.4	17.7	18.0	11.0–38.3	25.0	26.0
α-ZAL	<2–3.92	<2	<2	<2–3.50	<2	<2	<2–4.30	<2	<2
β-ZAL	ND	ND	ND	ND	ND	ND	ND	ND	ND

ND: non-detected

**Table 3:** Estimated dietary intake (DI) of DONs (DON, 15-AcDON, and 3-AcDON) from eggs in different areas by the Chinese population determined by a point evaluation (μg/kg BW/day).

Area	Populations	Mean ± SD	97.5th Percentile	Maximum
**JS**	**Children**	0.052 ± 0.248	0.291	2.34
	**Adults**	0.022 ± 0.114	0.121	0.968
	**Elder adults**	0.024 ± 0.128	0.135	1.09
**ZJ**	**Children**	0.039 ± 0.059	0.222	0.299
	**Adults**	0.016 ± 0.024	0.092	0.124
	**Elder adults**	0.018 ± 0.027	0.103	0.139
**SH**	**Children**	0.057 ± 0.068	0.194	0.253
	**Adults**	0.024 ± 0.028	0.081	0.105
	**Elder adults**	0.027 ± 0.031	0.090	0.117

**Table 4:** Estimated dietary intake (DI) of ZEN from eggs in different areas by the Chinese population determined by a point evaluation (μg/kg BW/day).

Area	Populations	Mean ± SD	97.5th Percentile	Maximum
**JS**	**Children**	0.021 ± 0.061	0.188	0.425
	**Adults**	0.009 ± 0.025	0.078	0.178
	**Elder adults**	0.009 ± 0.028	0.087	0.199
**ZJ**	**Children**	0.027 ± 0.134	0.156	0.853
	**Adults**	0.011 ± 0.056	0.065	0.354
	**Elder adults**	0.013 ± 0.062	0.073	0.396
**SH**	**Children**	0.031 ± 0.087	0.334	0.400
	**Adults**	0.013 ± 0.036	0.139	0.168
	**Elder adults**	0.014 ± 0.04	0.156	0.186

**Table 5:** Estimated dietary intake (DI) of ZEN from chicken tissues by the Chinese population determined by a point evaluation (μg/kg BW/day).

Population	Mean ± SD	97.5th Percentile	Maximum
**Children**	0.009 ± 0.002	0.012	0.012
**Adults**	0.005 ± 0.001	0.007	0.007
**Elder adults**	0.004 ± 0.001	0.005	0.006

**Table 6:** Average dietary consumption of eggs and chicken tissue by the three populations and average body weight.

Populations	Eggs (g/day) (Mean ± SD)	Chicken Tissues (g/day) (Mean ± SD)	Body Weight (kg) (Mean)
**Children**	25.1 ± 8.0	3.4 ± 1.7	24.5
**Adults**	25.6 ± 1.0	4.9 ± 0.6	60.3
**Elder adults**	28.3 ± 2.4	3.6 ± 0.9	59.4

**Figure A3:**
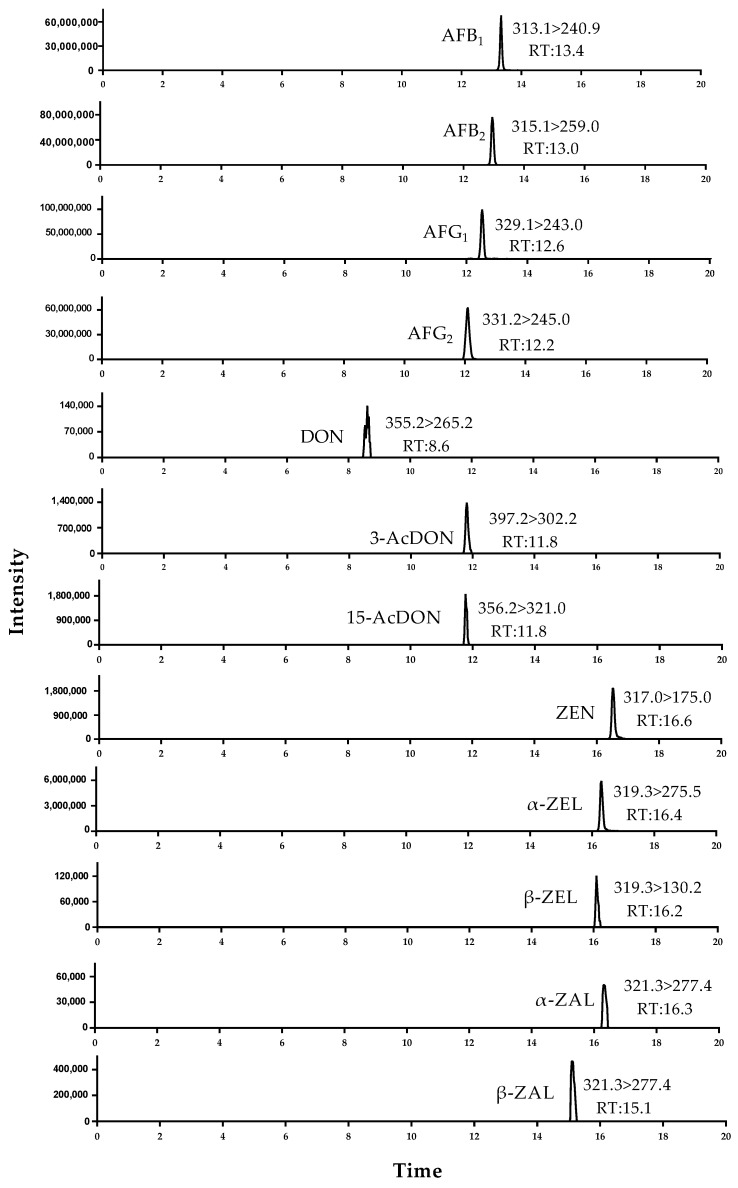
Chromatograms of 12 mycotoxins (blank tissue sample spiked at a different concentration of each analyte): AFB_1_ (25 ng/mL), AFB_2_ (25 ng/mL), AFG_1_ (25 ng/mL), AFG_2_ (25 ng/mL), DON (62.5 ng/mL), 3-AcDON (125 ng/mL), 15-AcDON (75 ng/mL), ZEN (31.25 ng/mL), α-ZEL (39 ng/mL), β-ZEL (39 ng/mL), α-ZAL (39 ng/mL), and β-ZAL (39 ng/mL).(RT: Retention Time)

## Appendix A

### Appendix A.1. Determination of Multimycotoxins in Chicken Tissues

#### Appendix A.1.1. Sample Pretreatment of Chicken Tissues

Tissue sample preparation was adapted from the method for pig tissue treatment published previously. Each tissue sample was homogenized for 120 s by a grinder. A total of 1.0 ± 0.05 g of the homogenized sample was weighed, mixed with 5 mL of water and 5 mL of acetonitrile (formic acid 0.1%), and shaken for 2 min. The system was subjected to extraction under ultrasonication for 30 min. MgSO_4_ (2.0 g) and NaCl (0.5 g) were added, followed by shaking for 2 min. After centrifugation for 5 min at 4000 rpm at room temperature, 1 mL of supernatant was mixed with 50 mg of C18 and 100 mg of MgSO_4_. The mixture was shaken vigorously for 2 min. After centrifugation at 4000 rpm for 5 min, the supernatant of the mixture was collected and then evaporated under a gentle stream of nitrogen gas at 50 °C. The residue was dissolved in 400 μL of acetonitrile/water (50:50, *v/v*) and mixed with 400 μL of hexane. After centrifugation at 5000 rpm for 5 min, the aqueous phase was collected and passed through a 0.22 μm nylon filter (Millipore, 13 mm diameter) for analysis.

#### Appendix A.1.2. LC-MS/MS Analysis

A liquid chromatography–tandem mass spectrometry system (Thermo, TSQ Vantage) was utilized for the simultaneous detection of mycotoxins. Chromatographic separation was achieved by an Agilent Extend-C_18_ column (100 mm × 4.6 mm, 3.5 μm) with a flow rate of 0.25 mL/min at 30 °C (4 °C for sampling room). The sample injection volume was 10 μL. The mobile phase was composed of phase A (water plus 5 mM ammonium acetate) and phase B (methanol). The elution program was set as follows: 5% B (initial), 5%–15% B (0–2 min), 15%–50% B (2–8 min), 50%–100% B (8–15 min), 100% B (15–20 min), 100%–5% B (22–22 min), and held a for a further 3 min for re-equilibration. The flow rate was 250 μL/min and the total run time was 25 min. A mass spectrometry analysis was carried out in both positive and negative ionization modes using multiple reaction monitoring (MRM). The following optimized conditions were used for the analysis: vaporizer temperature of 300 °C, ion transfer tube temperature of 350 °C, spray voltages of 3.5 KV (positive ESI) and 3 KV (negative ESI), sheath gas pressure of 30 psi, aux flow of 15 arb, collision gas (argon) pressure of 1.5 mTorr. Table A3 summarizes the optimized MS/MS parameters for the 12 mycotoxins in MRM mode.

#### Appendix A.1.3. LC-MS/MS Method Validation

The linearity, limit of detection (LOD), limit of quantification (LOQ), repeatability, and reproducibility of the 12 mycotoxins in different chicken tissues are presented in Table A4. Signal suppression and enhancement (SSE) was calculated by the following equation:

SSE = A/C × 100 (A1)

where A is the slope of the calibration curve in the matrix and C is the slope of the calibration curve in the neat solvent. Generally, SSE is considered tolerable when it varies from 80% to 120%. As demonstrated in Table A4, there is no obvious matrix effect. The results also show a good linear response between 0.936 and 0.999 with their linear ranges. The repeatability (RSD_r_) was determined in triplicate at three concentration levels on the same day, whereas the interday precision (RSD_R_) was calculated by analyzing the samples for three continuous days at three different concentration levels. The RSD (<20%) demonstrated the stability of the analytical method. The LOD and LOQ of the DONs were 2 and 5 μg/kg, while those of the other mycotoxins were 1 and 2 μg/kg. The selectivity of the method was investigated to evaluate the matrix interference at the retention time in the blank samples. Figure A3 shows chromatograms of 12 mycotoxins (blank tissue sample spiked at a different concentration of each analyte).

**Table A1:** Estimated dietary intake (DI) of DONs and ZEN from eggs by the Chinese population determined by the Monte Carlo assessment model (μg/kg BW/day).

Area	Toxins	Populations	Mean	50th Percentile	60th Percentile	70th Percentile	80th Percentile	90th Percentile	95th Percentile
**JS**	DONs	Children	0.054	0.039	0.05	0.063	0.082	0.115	0.147
		Adults	0.022	0.016	0.02	0.026	0.034	0.047	0.061
		Elder adults	0.025	0.018	0.023	0.029	0.038	0.053	0.068
	ZEN	Children	0.022	0.015	0.020	0.026	0.035	0.050	0.065
		Adults	0.009	0.006	0.008	0.010	0.015	0.021	0.027
		Elder adults	0.010	0.007	0.009	0.012	0.016	0.023	0.030
**JZ**	DONs	Children	0.039	0.029	0.037	0.046	0.059	0.082	0.104
		Adults	0.016	0.012	0.015	0.019	0.025	0.034	0.043
		Elder adults	0.018	0.013	0.017	0.021	0.027	0.038	0.048
	ZEN	Children	0.030	0.021	0.027	0.036	0.048	0.069	0.090
		Adults	0.013	0.009	0.011	0.015	0.020	0.029	0.037
		Elder adults	0.014	0.010	0.013	0.017	0.022	0.032	0.041
**SH**	DONs	Children	0.058	0.042	0.054	0.069	0.09	0.126	0.162
		Adults	0.024	0.017	0.022	0.028	0.037	0.052	0.067
		Elder adults	0.027	0.019	0.025	0.032	0.041	0.058	0.074
	ZEN	Children	0.031	0.025	0.029	0.038	0.050	0.072	0.093
		Adults	0.013	0.010	0.012	0.016	0.021	0.030	0.039
		Elder adults	0.014	0.012	0.013	0.017	0.023	0.033	0.043

**Table A2:** Estimated dietary intake (DI) of ZEN from chicken tissues by the Chinese population determined by the Monte Carlo assessment model (μg/kg BW/day).

Toxin	Populations	Mean	50th Percentile	60th Percentile	70th Percentile	80th Percentile	90th Percentile	95th Percentile
ZEN	Children	0.009	0.009	0.010	0.010	0.010	0.010	0.011
	Adults	0.006	0.005	0.005	0.006	0.006	0.007	0.007
	Elder adults	0.004	0.004	0.004	0.004	0.005	0.005	0.005

**Table A3:** Liquid chromatography–tandem mass spectrometry parameters of the 12 mycotoxins in chicken tissues in multiple reaction monitoring (MRM) mode.

Toxins	Precursor Ion (*m*/*z*)	Product Ions (*m*/*z*)	Collision Energy (eV)	Retention Time (min)	Ratio ^2^ (%)
DON	355.2 [M + CH_3_COO]^−^	265.2 ^1^ 247.2	10.0 14.0	8.6	35.68
3-AcDON	397.2 [M + CH_3_COO]^−^	307.2 ^1^ 173.2	16.0 17.0	11.8	54.81
15-AcDON	356.2 [M + NH_4_]^+^	321.0 ^1^ 136.8	–14.0 –15.0	11.8	41.40
AFB_1_	313.1 [M + H]^+^	240.9 ^1^ 284.9	–37.0 –26.0	13.4	95.11
AFB_2_	315.1 [M + H]^+^	259.0 ^1^ 287.1	–31.0 –29.0	13.0	90.39
AFG_1_	329.1 [M + H]^+^	243.0 ^1^ 214.0	–30.0 –40.0	12.6	87.69
AFG_2_	331.2 [M + H]^+^	245.0 ^1^ 189.0	–33.0 –45.0	12.2	82.99
ZEN	317.0 [M − H]^−^	175.0 ^1^ 273.0	25.0 20.0	16.6	72.78
α-ZEL	319.3 [M − H]^−^	275.5 ^1^ 130.3	20.0 38.0	16.4	31.29
β-ZEL	319.3 [M − H]^−^	130.2 ^1^ 160.3	34.0 30.0	16.2	75.17
α-ZAL	321.3 [M − H]^−^	277.4 ^1^ 303.4	22.0 22.0	16.3	32.14
β-ZAL	321.3 [M − H]^−^	277.5 ^1^ 303.4	22.0 22.0	15.1	32.14

^1^ Quantifying ion; ^2^ defined as the peak area of qualifier in percent of quantifier.

**Table A4:** Quality control parameters in the method for determining the 12 mycotoxins in hen tissues. SSE, signal suppression and enhancement; RSD_r_, repeatability; RSD_R_, interday precision; LOQ, limit of quantification; LOD, limit of detection.

Toxin	Linear Range (ng/mL)	R^2^	SSE (%)	RSD_r_	RSD_R_	LOQ (μg/kg)	LOD (μg/kg)
DON	5–1000	0.936	92.8	9.37	7.60	5	2
3-AcDON	5–600	0.969	75.1	2.82	4.82	5	2
15-AcDON	5–1000	0.983	66.1	2.61	1.92	5	2
AFB_1_	2–100	0.999	80.4	8.32	4.74	2	1
AFB_2_	2–100	0.998	77.4	17.58	8.08	2	1
AFG_1_	2–100	0.983	85.1	11.23	7.32	2	1
AFG_2_	2–100	0.995	103.3	7.26	8.47	2	1
ZEN	2–1000	0.999	74.5	4.49	11.08	2	1
α-ZEL	2–1000	0.999	87.25	3.31	15.39	2	1
β-ZEL	2–1000	0.999	81.7	5.13	11.09	2	1
α-ZAL	2–1000	0.997	89.4	2.47	13.35	2	1
β-ZAL	2–1000	0.999	81.5	6.07	6.61	2	1
